# General Practitioners’ Attitudes towards Essential Competencies in End-of-Life Care: A Cross-Sectional Survey

**DOI:** 10.1371/journal.pone.0170168

**Published:** 2017-02-01

**Authors:** Stéphanie Giezendanner, Corinna Jung, Hans-Ruedi Banderet, Ina Carola Otte, Heike Gudat, Dagmar M. Haller, Bernice S. Elger, Elisabeth Zemp, Klaus Bally

**Affiliations:** 1 Centre for Primary Health Care, University of Basel, Basel, Switzerland; 2 Institute for Biomedical Ethics, University of Basel, Basel, Switzerland; 3 Hospiz im Park, Hospital for Palliative Care, Arlesheim, Basel District, Switzerland; 4 Primary Care Unit, University of Geneva, Geneva, Switzerland; 5 Department of General Practice, the University of Melbourne, Melbourne, Victoria, Australia; 6 Center of Legal Medicine, University of Geneva, Geneva, Switzerland; 7 Swiss Tropical and Public Health Institute, University of Basel, Basel, Switzerland; 8 University of Basel, Basel, Switzerland; University of Ottawa, CANADA

## Abstract

**Background:**

Identifying essential competencies in end-of-life care, as well as general practitioners’ (GPs) confidence in these competencies, is essential to guide training and quality improvement efforts in this domain.

**Aim:**

To determine which competencies in end-of-life care are considered important by GPs, to assess GPs’ confidence in these competencies in a European context and their reasons to refer terminally ill patients to a specialist.

**Design and Setting:**

Cross-sectional postal survey involving a stratified random sample of 2000 GPs in Switzerland in 2014.

**Method:**

Survey development was informed by a previous qualitative exploration of relevant end-of-life GP competencies. Main outcome measures were GPs’ assessment of the importance of and confidence in 18 attributes of end-of-life care competencies, and reasons for transferring care of terminally-ill patients to a specialist. GP characteristics associated with main outcome measures were tested using multivariate regression models.

**Results:**

The response rate was 31%. Ninety-nine percent of GPs considered the recognition and treatment of pain as important, 86% felt confident about it. Few GPs felt confident in cultural (16%), spiritual (38%) and legal end-of-life competencies such as responding to patients seeking assisted suicide (35%) although more than half of the respondents regarded these competencies as important. Most frequent reasons to refer terminally ill patients to a specialist were lack of time (30%), better training of specialists (23%) and end-of-life care being incompatible with other duties (19%). In multiple regression analyses, confidence in end-of-life care was positively associated with GPs’ age, practice size, home visits and palliative training.

**Conclusions:**

GPs considered non-somatic competencies (such as spiritual, cultural, ethical and legal aspects) nearly as important as pain and symptom control. Yet, few GPs felt confident in these non-somatic competencies. These findings should inform training and quality improvement efforts in this domain, in particular for younger, less experienced GPs.

## Introduction

The European Association for Palliative Care recently described the core competencies of palliative care encompassing health professionals’ abilities to meet patient’s physical, social, psychological and spiritual needs [1]. However, most of the existing quality indicators of palliative care fall within the domain of physical care [2]. As a consequence, relatively little is known about health professionals’ attitudes towards a broad range of end-of-life care (EOLC) competencies. Since general practitioners (GPs) play an increasingly important role in palliative and end-of-life care their perspective is particularly important [3, 4].

In this context, a clear understanding of what GPs consider important at the end of life is crucial. About a decade ago, pain and symptom management, doctor-patient communication, preparation for death, and the opportunity to achieve a sense of completion was rated as important by most GPs [5, 6]. However, GPs’ educational preferences in palliative care mainly encompassed symptom control such as opiate prescription, the control of nausea and vomiting and the use of a syringe driver [7].

The UK’s Gold Standards Framework (GSF) is a model that enables good practice to be available to all people nearing the end of their lives, irrespective of diagnosis [8]. One of the five goals of GSF is to develop increased competence and confidence in end-of-life care. Confidence in the ability to provide palliative care is an essential prerequisite for providing end-of-life care. Its absence might lead to hesitations in providing end-of-life care at all. There is evidence that GPs were more likely to feel confident with increasing experience of home care and of caring for cancer patients [9] as well as with a higher number of palliative trajectories for which they had been responsible [10].

Furthermore, systemic barriers to provision of palliative care among GPs need to be identified and addressed [11, 12]. In Australia, the percentage of GPs refusing to provide palliative care was shown to be as high as 25% [13]. Most frequently reported reasons for not providing palliative care include that GPs do not do home visits, that they feel there is inadequate support, that they have family or personal commitments or because of lack of knowledge [13].

In order to adequately inform current GPs’ training in palliative care and to guide quality improvement efforts, the current study aimed to explore GPs’ attitudes towards a broad range of end-of-life care competencies such as symptom management, ethical, legal, cultural and spiritual aspects. In particular, we focused on GPs’ assigned importance of and confidence with end-of-life care competencies how they relate to each other and to GPs’ characteristics. Furthermore, we aimed to assess GPs’ reasons to transfer terminally ill patients to a specialist.

## Materials and Methods

### Ethics approval

The design of the study and participants’ selection were approved by the ethical review committee of Basel (Nordwest- und Zentralschweiz Nr. EK 248/12) in January 2013 and January 2014, respectively. In the study information, we informed the participants that there will be no tracking of any identifying information and that the responses will be completely anonymous. Since the survey was anonymous it could be assumed that GPs who responded to the survey agreed with participation in the survey. Therefore GPs were not required to sign an informed consent form.

### Study design and setting

A nationwide, questionnaire-based survey was conducted among GPs covering various language regions (German-/French-/Italian-speaking) of Switzerland. The survey was part of an observational, cross-sectional study entitled “Conditions and Quality of End-of-Life Care in Switzerland—the role of general practitioners” to assess GPs’ attitudes towards EOLC.

### Sampling and data collection

A representative, stratified random sample of 2000 GPs was selected from the official register of the Swiss Medical Association (FMH) in February 2014 [14]. Stratification was based on language region, gender and age [15, 16]. Since the survey was anonymous, the questionnaire was mailed to all participants twice; in March 2014 and a reminder in April 2014. The letter included the study information, a questionnaire and a stamped addressed envelope to return the study documents (see S1 Appendix).

### Instrument development

After an exploratory approach on existing relevant quality indicators for specific target areas of palliative care [17, 18], key factors in care quality were assessed via 23 qualitative interviews with GPs, 7 interviews with relatives of palliative patients, 3 interviews with palliative patients and 3 focus groups involving 10 health care professionals from each language region of Switzerland. The questionnaire was based on results of this qualitative study part. It was developed in German and tested by 10 GPs to confirm its face validity and ease of application. After the pre-test and final adjustments, the questionnaire was translated into French and Italian. The order of administration of items covering importance of different EOLC, confidence in these competencies and reasons for transfer of terminally ill patients is listed in the Supporting Information (see S2 Appendix). For the current study, the following parts of the questionnaire were used:

#### Attitudes towards different EOLC quality criteria

The importance of and confidence in different EOLC quality criteria were assessed from a total set of 20 items each of which the content of 18 items overlapped (see S2 Appendix). All items used a 5-point Likert scale ranging from “unimportant” to “very important” and from “unconfident” to “very confident”. The 18 overlapping items of the multi-item scale were added to a sum score for each GP and averaged to form a mean score representing overall EOLC confidence and assigned importance.

#### Reasons to transfer terminally ill patients to a specialist

The questionnaire included 7 items related to the reasons to transfer terminally ill patients to specialists, using a 5-point Likert scale ranging from “strongly disagree” to “strongly agree”. Point values from the 7 items of the multi-item scale were added to create a sum score for each GP and averaged to obtain a mean score representing overall agreement to transfer terminally ill patients to specialists.

#### Demographic, geographic and professional characteristics

The questionnaire further assessed demographic characteristics (gender and age), geographic characteristics (urban/rural context of practice site, number of inhabitants and the spoken language of the region) and professional characteristics (practice size, workload, number of consultations per half day, number of home visits per month, number of deceased patients for whom GPs were the main doctor in the previous year (2013) and the completion and duration of vocational palliative care training).

### Statistical analyses

First, descriptive statistics are reported for demographic, geographic and professional characteristics of GPs. Descriptive analyses also included frequencies and percentages of GPs’ attitudes towards EOLC. For descriptive parsimony in the main text, we collapsed the two highest point values of the attitudes towards different EOLC quality criteria and reasons to transfer terminally ill patients to a specialist into one: e.g. “fully agree” and “agree” into “agree”. Frequencies of each category are reported in the Supporting Information (see S1, S2 and S3 Tables). Item-level analyses providing item-total correlations, standard deviations in item and reliability indices were conducted using the R package”psychometric” [19]. Item correlation matrices using Spearman’s rank correlation coefficient were performed using R package “psych” [20]. Additionally, the correlation between GPs’ confidence and assigned importance in EOLC competencies averaged across GPs was assessed. For scale-level analysis, composite scores across the multi-item scales were computed (mean scores). After testing for normality of data using Shapiro-Wilk test, the difference between the mean ranks of the composite scores of GPs’ confidence and importance across all EOLC competencies was assessed using Wilcoxon signed-rank test. Reliability indices of the multi-item scales were examined using Cronbach’s alpha and coefficient omega [21]. Omega makes fewer and more realistic assumptions than alpha. In particular, omega was shown to outperform alpha under violations of tau-equivalence [22]. Moreover, the calculation of Omega with a confidence interval (CI) better reflects the variability of a population [23, 24]. Thus, we provide coefficient omega with 95% confidence intervals using bootstrapping with n = 1000 simulations [25].

#### Multivariate regression analysis

The associations of demographic and professional characteristics with the dependent variables were investigated by multivariable adjusted robust linear regression analyses [26–28]. Dependent variables were the overall confidence (mean score) and the overall agreement with reasons to transfer terminally ill patients to a specialist (mean score). Multivariable adjusted regression estimates (β) and their 95% confidence intervals (CI) are reported. Age category, gender, workload, practice size, number of consultations per half day, number of home visits per month and the completion of palliative training were included as covariates of interest in all models. In addition, level of confidence with EOLC competencies was included in the model on agreement with reasons to refer terminally ill patients. Language region and urban context of GP practices were included in the model as confounders. P values of < 0.05 were considered statistically significant. All analyses were completed using R 3.13 [29].

## Results

### Participant demographics

**Fig 1:**
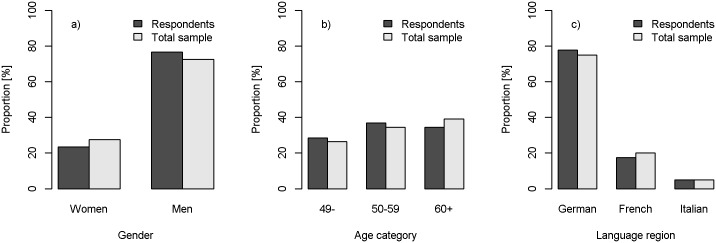
Comparison of respondents (N = 579) and total sample (N = 2000) by gender, age and language region.

Out of the address list of 2000 physicians from the FMH, 7 were not eligible because they were no longer residents of the community. Thus, 1993 general practitioners in three language regions of Switzerland were invited to participate in the survey. 100 physicians reported that they were no longer working as GPs and were excluded. From the total of 1893 eligible GPs, 579 sent back a questionnaire (response rate = 31%). There was no significant difference in age category (*χ*^*2*^ = 3.96, p = 0.13), gender (*χ*^*2*^ = 3.74, p = 0.05) or language region of the practice (*χ*^*2*^ = 1.97, p = 0.37) between GPs who responded to the survey and the total sample of eligible GPs (see Fig 1).

Table 1 summarizes the characteristics of GPs who responded to the survey. The majority of respondents were between 50 and 59 years old (37%) and male (77%). 14% of GPs had undergone specific vocational training in palliative care. The percentage of GPs who were principally responsible for at least one palliative patient within the last year was 80% for tumor patients and 82% for non-tumor patients.

**Table 1:** Demographic and professional characteristics of GPs (N = 579).

Characteristics	n^a^ (%), unless otherwise stated
Age (years)	
≤49	165 (29%)
50–59	214 (37%)
≥ 60	200 (35%)
Male sex	443 (77%)
Single practice	261 (45%)
Median percent per position (SD)	100 (26)
Median number of consultations per half-day (SD)	13 (5)
Median number of home visits per month (SD)	6 (12)
Specific vocational palliative care training	81 (14%)
Number of deceased cancer patients for whom GP was main doctor in the past year	
0	111 (19%)
1–5	410 (71%)
≥6	50 (9%)
Number of deceased non-cancer patients for whom GP was main doctor in the past year	
0	96 (17%)
1–5	353 (61%)
≥6	123 (21%)

^a^ Missing values for Gender, n = 1(0.2%), Workload in percent, n = 3 (0.5%), Practice type, n = 9 (1.6%), Consultations per half-day, n = 3 (0.5%), Home visits per month, n = 16 (2.8%), Specific vocational training in palliative care, n = 1 (0.2), Number of deceased cancer patients for whom GP was main doctor in the past year, n = 8 (1.4%), Number of deceased non-cancer patients for whom GP was main doctor in the past year, n = 7 (1.2%).

### Item-level analysis

#### Importance of different quality criteria of EOLC

**Fig 2:**
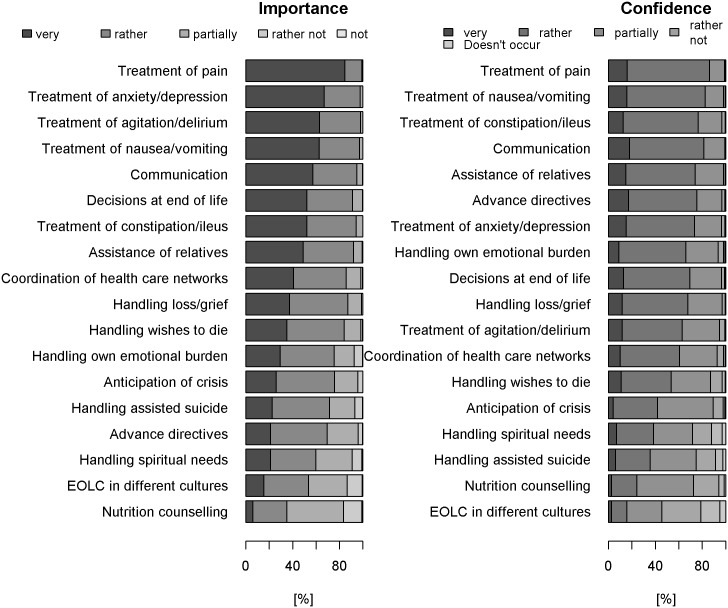
Ranked EOLC competencies. EOLC competencies were ranked according to their importance (left) and the level of confidence GPs have in these competencies (right).

EOLC quality criteria, sorted according to GPs’ assessment of importance, are displayed in Fig 2. **Symptom management:** Almost all GPs considered the recognition and treatment of pain as an important criterion of EOLC quality (99%). Likewise, recognition and treatment of anxiety/depression (98%), agitation/delirium (98%), nausea/vomiting (97%) and the treatment of constipation/ileus (94%) were reported as important. **Ethical and legal aspects:** Most GPs stated that handling decisions at the end-of-life (91%), advance directives (69%), patients seeking assisted suicide (72%) and wishes to die (84%) were important criteria for end-of-life care quality. **Cultural aspects:** End-of-life care of patients of different cultures was acknowledged as important by half of respondents (53%). **Spiritual aspects:** Nearly two thirds of GPs stated that handling spiritual needs was an important EOLC quality criterion (60%). **Communication:** Communicative competencies were important for the vast majority of GPs (95%). Further descriptive statistics and the frequencies of all response categories for all items about importance of EOLC competencies as well as the item-level analysis can be found in the Supporting Information (see S1 Dataset, S1 and S4 Tables).

#### Confidence in different EOLC competencies

**Fig 3:**
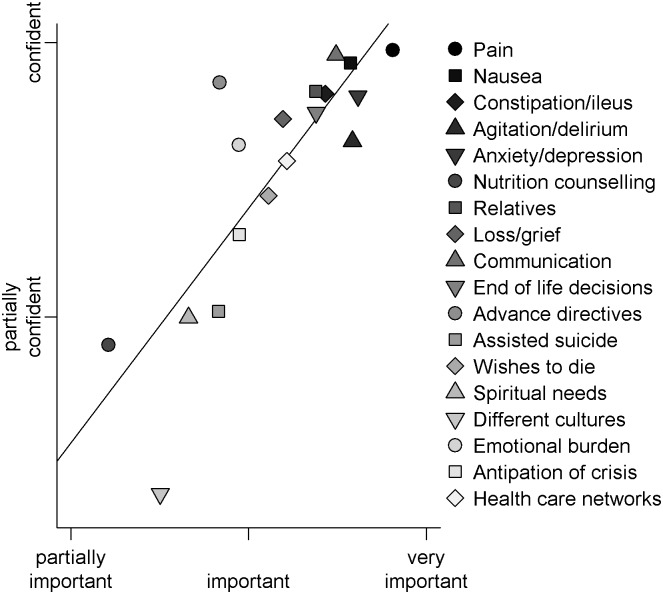
Plot of association between assigned importance and confidence levels of different EOLC competencies averaged across GPs. The x-axis describes the level of importance and the y-axis the level of confidence of GPs. For each of the 18 EOLC competencies the mean importance and confidence values across GPs are displayed.

EOLC competencies, sorted according to GPs confidence in providing these competencies, are displayed in the right column of Fig 2. **Symptom management:** The majority of GPs felt confident in the recognition and treatment of pain (86%), anxiety/depression (73%), agitation/delirium (63%), nausea/vomiting (83%) and constipation/ileus (77%). **Ethical and legal aspects:** Most GPs felt confident in handling decisions at the end-of-life (70%) and advance directives (75%). Clearly less GPs felt confident in handling patients seeking assisted suicide (35%) and the wish to die (54%). **Cultural aspects:** Only a small proportion of GPs were confident in palliative care of patients with different cultural background (16%). **Spiritual aspects:** In handling spiritual needs a third of GPs reported they felt confident (38%). **Communication:** Most GPs felt confident in EOLC communication (81%). Further descriptive statistics and the frequencies of all response categories for all items about confidence in EOLC as well as the item-level analysis can be found in the Supporting Information (see S2 and S5 Tables). The correlation between confidence and importance levels for different EOLC competencies averaged across GPs was significant and can be seen in Fig 3 (Pearson’s R = 0.83 (95% CI: 0.58, 0.93), p < 0.001).

#### Reasons to refer terminally ill patients to a specialist

**Fig 4:**
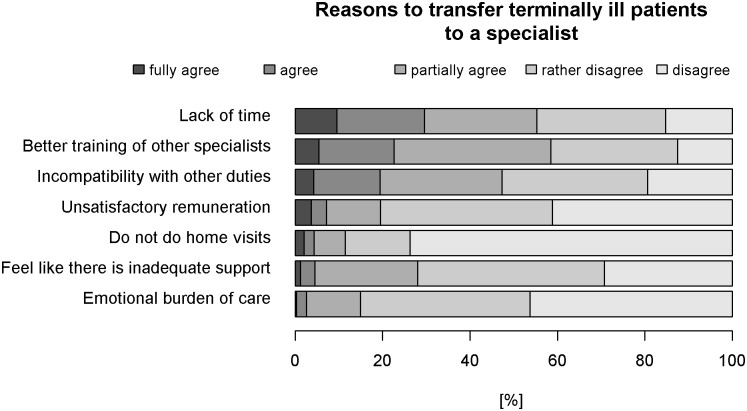
Ranked reasons for transferring terminally ill patients to specialists. The reasons for transferring terminally ill patients to specialists are ranked according to the proportion of GPs who agreed with this reason.

Reasons for referring terminally ill patients to a specialist are shown in Fig 4, sorted by GPs’ agreement with each item. Lack of time (30%), better training of other specialists (23%) and incompatibility with other duties (19%) were most often reported reasons for referring terminally ill patients definitively to a specialist. Further descriptive statistics and the frequencies of all response categories for all items as well as the item-level analysis can be found in the Supporting Information (see S3 and S6 Tables).

### Scale-level analysis

Internal consistency of the 18 items covering GPs’ confidence across EOLC competencies was good with a raw Cronbach’s alpha of 0.89 and a coefficient omega of 0.89 (95% CI: 0.87, 0.90). Average inter-item correlation was 0.32 across items covering confidence in EOLC competencies (see S7 Table for the correlation matrix). The multi-item scale of the 18 items covering importance across EOLC competencies showed good to excellent internal consistency with a Cronbach alpha of 0.89 and coefficient omega of 0.90 (95% CI: 0.88, 0.91). Average inter-item correlation was 0.32 (see S8 Table for the correlation matrix). The raw Cronbach’s alpha for the 7 items covering reasons to refer terminally ill patients to specialists was 0.78 and coefficient omega was 0.79 (95% CI: 0.76, 0.82) indicating acceptable internal consistency. Average inter-item correlation was 0.36 across items covering reasons to refer terminally ill patients to specialists (see S9 Table for the correlation matrix). Descriptive statistics for the multi-item scales can be found in the Supporting Information (see S10 Table).

#### Comparison between mean scores of importance and confidence in different EOLC competencies

**Fig 5:**
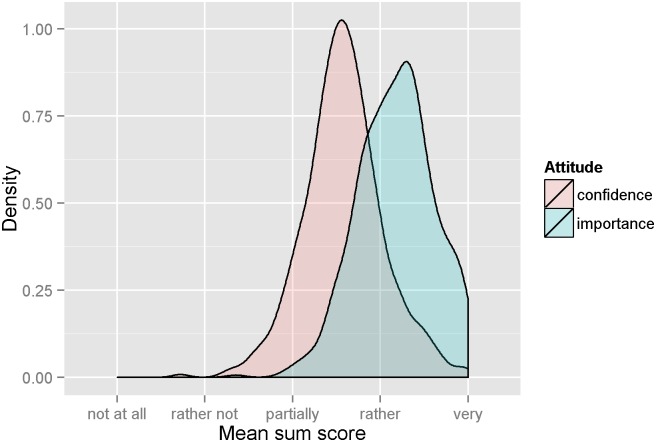
Density plot of mean score of GPs’ assigned confidence and importance across all EOLC competencies. Point values from all 18 items assessing importance of and confidence in different EOLC quality criteria were added to a sum score for each GP and averaged to a mean score to represent overall EOLC confidence and assigned importance.

Importance across different EOLC competencies was significantly higher (median = 4.22, median absolute deviation = 0.41, see Fig 5 and S10 Table) than confidence across different EOLC competencies (median = 3.56, median absolute deviation = 0.41) (Wilcoxon signed-rank test = 102273, p < 0.0001).

### Multivariate analyses

#### Confidence in EOL care

Results from multivariate analyses, controlling for confounders (see Table 2), showed that older GPs were significantly more likely than younger GPs to feel confident about EOLC competencies (*β* = 0.17, 95%CI: 0.07 0.27). GPs who paid more home visits and who had completed a specific vocational training were more likely to feel confident (*β* per 10 monthly home visits = 0.08, 95%CI: 0.05 0.11, *β* = 0.10, 95% CI: 0.00, 0.20). Likewise, GPs working in a group practice were more likely to feel confident than GPs working in a single practice (*β* = 0.13, 95%CI: 0.04–0.21). The findings are presented in Table 2.

**Table 2:** Association between GP characteristics and overall confidence across different EOLC competencies.

	β		95% CI	p
Age(ref.<50)				
50–59	0.05	-0.04	0.15	0.290
60+	0.17	0.07	0.27	0.001
Male Gender (ref. female)	0.05	-0.06	0.15	0.397
Workforce (per day)	0.01	-0.03	0.05	0.610
No. of consultations per half day	0.00	-0.01	0.01	0.410
No. of home visits per month (per 10 home visits)	0.08	0.05	0.11	0.000
Group practice (ref. Single)	0.13	0.04	0.21	0.003
Palliative training (ref. no training)	0.10	0.00	0.20	0.059
Language(ref. German)				
French	-0.12	-0.22	-0.01	0.027
Italian	0.02	-0.14	0.19	0.783
Urban (ref. city)				
Sub-urban	-0.04	-0.14	0.06	0.443
Rural/Mountain	-0.04	-0.13	0.05	0.388

Results from multiple linear regression analysis, outcome: mean score across confidence in EOLC.

#### Agreement about reasons to refer terminally ill patients to a specialist

Results from multivariate analyses, controlling for confounders (see Table 3), showed that GPs older than 60 years were significantly less likely to transfer terminally ill patients to a specialist than GPs younger than 50 years (*β* = -0.24, 95%CI: -0.39–0.08). GPs with higher confidence across different EOLC competencies and with a higher number of patient consultations per half day were less likely to refer terminally ill patients to a specialist (*β* per point Likert scale increase = -1.65 95%CI: -2.34–0.96, *β* per consultation = -0.02 95%CI: -0.03–0.001). The findings are presented in Table 3.

**Table 3:** Association between GP characteristics and overall agreement with reasons to refer terminally ill patients to a specialist.

	β		95% CI	p
Confidence (per Likert scale point)	-1.65	-2.34	-0.96	0.000
Age(ref.<50)				
50–59	-0.04	-0.17	0.09	0.553
60+	-0.24	-0.39	-0.08	0.002
Male Gender (ref. female)	-0.08	-0.24	0.07	0.300
Workforce (per day)	0.03	-0.03	0.09	0.281
No. of consultations per half day (per consultation)	-0.02	-0.03	0.00	0.046
No. of home visits per month (per 10 home visits)	-0.13	-0.27	0.02	0.097
Group practice (ref. Single)	0.04	-0.08	0.17	0.475
Palliative training (ref. no training)	-0.10	-0.24	0.03	0.144
Language region (ref. German)				
French	0.32	0.17	0.47	0.000
Italian	-0.13	-0.41	0.14	0.346
Urban (ref. city)				
Sub-urban	-0.04	-0.19	0.10	0.576
Rural/mountain	-0.15	-0.28	-0.03	0.015

Results from multiple linear regression analysis, outcome: mean score across agreement reasons to refer terminally ill patients to specialists.

## Discussion

The majority of GPs felt non-somatic competencies (e.g. legal, ethical, cultural and spiritual competencies) were nearly as important as somatic competencies (e.g. pain and symptom control). Yet, they felt less confident in managing legal, ethical, cultural and spiritual aspects of end-of-life care. Older age, a higher number of home visits and the completion of palliative care training were positively associated with GPs’ confidence to provide palliative care. Lack of time, better training of specialists and incompatibility with other duties were the most frequently reported reasons for referring terminally ill patients to a specialist.

GPs considered symptom and pain control as the most important palliative care competencies, which supports previous literature findings and is underscored by GPs’ educational preferences [5, 7, 18]. The finding that more than half of the respondents also considered the competencies in spiritual, cultural, ethical and legal aspects as important EOLC quality criteria is in line with the core competencies outlined by the European Association for Palliative Care [1] and the Gold Standards Framework [8].

The current study showed that GPs’ overall confidence across EOLC competencies was lower than their assigned importance to EOLC competencies. The average of the composite score ranged between partially confident and confident on the Likert scale. This is in line with the findings from a Danish study stating that a majority of Danish GPs were moderately confident being principally responsible for a palliative care trajectory [10]. Australian GPs have also been shown to be least confident about psychosocial problems and technical aspects of palliative medicine [7]. We observed on the one hand a positive association between GPs’ age as well as GPs’ number of home visits with their level of confidence in palliative care. On the other hand, we found a negative association between GPs’ palliative care confidence as well as their age and their tendency to refer terminally ill patients to a specialist. These findings might indicate a “learning by doing” effect in end-of-life care confidence. In accordance, it has been shown that GPs palliative care confidence was positively associated with the number of palliative trajectories for which the GP had been responsible [10] and that accumulated skills were a facilitator in GPs engagement in advance care planning [30].

A strong association was found between GPs assigned importance and confidence across different EOLC competencies (see Fig 3). Intriguingly, the average confidence level of GPs to treat patients of different cultures was lower than expected by their level of assigned importance (see Fig 5). This is not surprising considering the diverse attitudes towards death and dying across cultures which can range from stigma/taboo to medicalization and accumulation of curative procedures [31]. Indeed, there is little literature on how cultural differences might affect what is considered appropriate care for patients at the end of life and how these issues are handled across countries [32]. In particular, there is an absence of robust research reporting or evaluating end of life interventions for people of non-dominant cultures, which would be necessary to deliver culturally safe palliative care [33]. As a result, the European research agenda for cultural issues in EOLC research recently decided to focus on culture, diversity, and their operationalization [34].

Spirituality is a major domain of palliative care training to ensure that patients can find meaning and hope even in the last period of their life [35, 36]. However, the current results showed that GPs only felt partially confident in handling spirituality in EOLC. Furthermore, there is little literature on the methods or contents of spiritual training for GPs. A qualitative evidence synthesis study indicated that GPs would like to support patients’ spiritual wellbeing, but lack specific skills and attitudes to assess spiritual needs and to provide spiritual care [37]. Regarding the evidence that spiritual care training has a positive influence on the spiritual well-being and the attitudes of the participating palliative care professionals [38], GPs might benefit from additional support and training in spiritual caring practice in palliative care.

The current study further showed that GPs displayed lower confidence in ethical and legal aspects compared to pain and symptom management. This is the case especially for handling patients seeking assisted suicide, which is in line with another Swiss study indicating that more than 50% of GPs had never been confronted with a request for assisted suicide and would rather hesitate in this situation [39]. In comparison, an Irish study indicated that 30% of respondent GPs have received requests from patients for euthanasia in the past five years and only 10% of GPs would be willing to participate in physician-assisted suicide [40]. In the Netherlands which was the first country to legally permit euthanasia or physician-assisted suicide, 44% of all explicit requests for euthanasia or physician-assisted suicide directed at GPs resulted in euthanasia or physician-assisted suicide [41]. A Dutch study further indicated that GPs would generally like to avoid requests for euthanasia but half of them were nevertheless open to consider a patients’ request for assisted suicide if the suffering of a patient could not be lessened [42].

The number of home visits that GPs make in a normal week varies considerably across Europe. A study of 2003 showed that GPs in Belgium provided most visits per week with an average of 44 visits per week while GPs from Portugal delivered only 2 home visits per week (44). By 2011, the median number of home visits per week carried out in Germany was 6.5 [43]. In comparison, the respondent GPs reported a median of 6 home visits per month, clearly ranging at the lower bound of delivering home visits across the European average. Delivering home visits is an important aspect of access for seriously ill or disabled patients who are unable to reach the office [44]. In particular, the availability of the GP for home visits and after office-hours was considered as crucial by patients and GPs for good end-of-life care [45]. The current results provide evidence that the number of home visits is positively associated with the confidence levels of GPs to provide EOLC. However, in most European countries and the US the number of home visits carried out by GPs has been decreasing continuously [46, 47]. In Netherlands, the proportion of home visits decreased from 14% in 1987 to 7% in 2001 [46]. Thus, the developments in the organisation of primary care such as the restriction of time for home visits, more part-time jobs and out-of-hours services may threaten valued aspects in end-of-life care [45].

In the current survey, GPs’ reasons to refer patients to a specialist most often encompassed lack of time, better training of specialist and incompatibility with other duties. In comparison, Australian GPs listed the absence of home visits, the lack of support and personnel commitments most often as reasons to refer terminally ill patients to a specialist [48]. Although the workload of Swiss GPs is rather low compared with other European countries [49], time constrains still seem to play an important role in GPs’ decision to refer terminally ill patients to a specialist. Regression results showed that younger GPs were more likely to agree to refer terminally ill patients to specialists. This finding might be based on generational differences in medical education and palliative care training as well as teaching styles in medicine [50]. While older GPs had a broader general medical training and were used to responding to a variety of health needs on their own, younger GPs were trained in a more specialist-centred professional environment and may be more likely to refer patients to specialists.

Moreover, there is evidence that primary care models may affect referral rates indicating that fee-for-service models are associated with lower referral rates compared to capitation models [51]. Interestingly, the majority of respondent GPs reported that remuneration was not a reason to refer terminally ill patients to specialists. This fits in with the primary health care system in Switzerland in which providers are typically paid on a fee-for-service basis unlike in the UK or in Germany. The current results also indicated that GPs with a higher number of patient consultations per half day were less likely to refer terminally ill patients to a specialist independently of age and confidence levels. This might relate to the finding that GPs operating in a more competitive market were shown to have higher referral rates since they may be more inclined to satisfy patients requests for referrals [52]. Furthermore, we found that older GPs would less often agree to refer patients to specialists. This finding might be based on generational differences in medical education and palliative care training as well as teaching styles in medicine [50]. While older GPs had a broader general medical training and were used to address a variety of health issues by themselves, younger GPs were trained in a more specialist-centred professional environment and may be more likely to refer patients to specialists.

The current study showed that confidence was lower than importance across EOLC competencies (see Fig 5). A study from the UK showed that reduced confidence in palliative care symptom control was associated with increased difficulty in accessing information on palliative care skills [7]. Although training for palliative care is rarely included in healthcare education curricula [53], the European Palliative Care Associations recommends 40 hours of palliative care training to achieve the goals of the curriculum in palliative care [54]. In Switzerland, undergraduate education for training in palliative medicine shows significant deficiencies compared to these recommendations [31, 55]. In 2007, Swiss undergraduate medical schools curricula provided an average of 10 hours of mandatory palliative care education [56], which increased up to 14.5 hours by 2012 [57]. Swiss undergraduate medical schools mainly focus on ethics-related contents (41%) and communication skills in palliative care are largely limited to breaking bad news. Continuing medical training on EOLC was shown to increase confidence in EOLC-related communication and collaboration skills in GPs [58] and to improve self-rated confidence, competence and knowledge in EOLC among hospital and community staff [59]. Thus, GPs may benefit from palliative care training as it is recommended by European Palliative Care Association’s guidelines.

A limitation of this study was the response rate of 31% which might be related to the length of the questionnaire. The total respondent burden was relatively high considering an average of 30 minutes for the completion of the questionnaire (total number of items n = 173). As the respondents did not differ from non-respondents in terms of age, gender and language region, we do not have any formal indication for a strong selection bias. We cannot exclude, however, that more GPs participated who were interested in or were particularly affected by the issue of EOLC. We believe that the main findings of our study are only partially affected by the response rate. Importance ratings of different EOLC competencies might be overestimated. However, if confidence levels are low in a very motivated sample, we would expect them to be even lower in a sample including less motivated physicians with lower interest in the topic. Therefore, despite the low response rate, our findings that GPs lack confidence in cultural, spiritual and legal EOLC competencies suggesting a particular need to support younger, less experienced GPs remains valid. A strength of this study was the quantitative assessment of GPs attitudes to a wide range of palliative care competencies.

Since confidence levels were significantly lower than the assigned levels of importance across different EOLC quality criteria, this survey clearly suggests a need for systematic palliative training of GPs, and the findings of the multivariate analyses imply that particularly the younger generations of GPs are to be targeted for such training. The training should not only encompass areas of pain and symptom control but also spiritual, cultural, ethical and legal domains of palliative care. Postgraduate training curricula should be revised to include the acquisition of EOLC competencies.

In view of upcoming demographic changes, health care structures need to be adapted to facilitate palliative care provided by GPs. However, further research is needed, especially about which EOLC competencies are important for patients, their relatives and their accompanying nurses as well as their opinion on the role of GPs in palliative care.

## Supporting Information

S1 Appendix Study information and questionnaire. Addressed to GPs upon receiving the questionnaire. Study information on page 1. Questionnaire pages 2–15.
(PDF)

S2 Appendix Sample survey items of EOLC competencies (translated to English). (DOCX)

S1 Dataset CSV File with the individual datapoints. The csv file contains all variables analysed within this study (demographic items (n = 11), EOLC confidence items (n = 18), EOLC importance items (n = 18) and reasons to transfer terminally ill patients to specialists (n = 7) of all 579 respondents. Missing data is indicated by NA.
(XLSX)

S1 Table Descriptive statistics of importance across different EOLC competencies. The descriptive statistic encompass number of valid cases (n), mean, standard deviation (sd), median (standard), minimum (min), maximum (max), skew, kurtosis, standard error (se). Furthermore, frequencies (percentages of responses in each category) for each response category are presented.
(XLSX)

S2 Table Descriptive statistics of confidence across different EOLC competencies. The descriptive statistic encompass number of valid cases (n), mean, standard deviation (sd), median (standard), minimum (min), maximum (max), skew, kurtosis, standard error (se). Furthermore, frequencies (percentages of responses in each category) for each response category are presented.
(XLSX)

S3 Table Descriptive statistics of reasons to refer terminally ill patients to specialists. The descriptive statistic encompass number of valid cases (n), mean, standard deviation (sd), median (standard), minimum (min), maximum (max), skew, kurtosis, standard error (se). Furthermore, frequencies (percentages of responses in each category) for each response category are presented.
(XLSX)

S4 Table Item level analysis of importance across different EOLC competencies. Provides item-total correlations (Item.total), item-total correlation scored without item (Item.Tot.woi), standard deviation in items (Sample.SD), reliability indices (Item.Reliab), reliability indices scored without item (Item.Rel.woi).
(XLSX)

S5 Table Item level analysis of confidence across different EOLC competencies. Provides item-total correlations (Item.total), item-total correlation scored without item (Item.Tot.woi), standard deviation in items (Sample.SD), reliability indices (Item.Reliab), reliability indices scored without item (Item.Rel.woi).
(XLSX)

S6 Table Item level analysis of reasons to refer terminally ill patients to a specialist. Provides item-total correlations (Item.total), item-total correlation scored without item (Item.Tot.woi), standard deviation in items (Sample.SD), reliability indices (Item.Reliab), reliability indices scored without item (Item.Rel.woi).
(XLSX)

S7 Table Correlation matrix of items covering confidence across different EOLC competencies. Spearman correlation coefficient was used.
(XLSX)

S8 Table Correlation matrix of items covering importance across different EOLC competencies. Spearman correlation coefficient was used.
(XLSX)

S9 Table Correlation matrix of items covering reasons to refer terminally ill patients to a specialist. Spearman correlation coefficient was used.
(XLSX)

S10 Table Descriptive statistics of mean score of confidence/importance across different EOLC competencies and reasons to refer terminally ill patients to a specialist. The descriptive statistic encompass number of valid cases (n), mean, standard deviation (sd), median (standard), median absolute deviation (from the median) minimum (min), maximum (max), skew, kurtosis, standard error (se).
(XLSX)
