# Supplementary material for: General Practitioners’ Attitudes towards Essential Competencies in End-of-Life Care: A Cross-Sectional Survey
Source: PLoS One. 2017 Feb 1;12(2):e0170168. doi: 10.1371/journal.pone.0170168 (PMC5287469; doi:10.1371/journal.pone.0170168)
Supplement: S1 Appendix — Addressed to GPs upon receiving the questionnaire. Study information on page 1. Questionnaire pages 2–15. (PDF) [file pone.0170168.s001.pdf]

5592417317

UNIVERSITÄT BASEL

Medizinische Fakultät

Betreuung von Patienten/Patientinnen am Lebensende
(End-of-Life Care) - die Rolle der Hausärzte/Hausärztinnen

Nationales Forschungsprogramm NFP 67 (www.nfp67.ch)

Institut für Hausarztmedizin der Universität Basel

Eine Kooperationsstudie in Zusammenarbeit mit allen Schweizerischen Instituten für Hausarztmedizin, Hausärzte Schweiz, dem Institut für Bio- und Medizinethik der Universität Basel, dem Hospiz im Park, Klinik für Palliative Care in Arlesheim, dem Institut für Kooperationsforschung und - entwicklung der FH Nordwestschweiz und dem Schweizerischen Tropen- und Public Health Institut der Universität Basel

Liebe Frau Kollegin, lieber Herr Kollege,

- Bitte antworten Sie der Reihe nach (mit Bleistift, Kugelschreiber oder Tinte) und **lassen Sie möglichst keine Frage aus. Mehrfachantworten sind nur dann möglich, wenn ausdrücklich vermerkt.**
- Falls keine Antwort vollständig zutrifft, antworten Sie bitte so, wie es **am ehesten für Sie stimmt**.
- Es gibt keine richtigen oder falschen Antworten. Nur Ihre **persönliche Meinung und Situation** ist von Interesse.
- **Wenn Sie nicht als Hausarzt/Hausärztin tätig sind**, kreuzen Sie bitte nur die erste Frage an und schicken Sie uns den gesamten Fragebogen ohne die weiteren Fragen zu beantworten im vorfrankierten Rückumschlag zurück.
- Die Auswertung der Daten erfolgt **vollständig anonymisiert**. Eine Zuordnung von Informationen zu einer bestimmten Person wird dem Studienteam nicht möglich sein.
- Bei Unklarheiten wenden Sie sich bitte an die Studienleitung (klaus.bally@unibas.ch oder ina.otte@unibas.ch)
- Bitte senden Sie uns den Fragebogen bis zum **31. März 2014** im vorfrankierten Rückumschlag zurück.

Wir danken Ihnen herzlich für Ihre Mühe und Unterstützung.

Dr. med. Klaus Bally
Facharzt für Allgemeine Medizin FMH
Institut für Hausarztmedizin der Universität Basel
St. Johanns-Parkweg 2
CH 4056 Basel
Tel: ++ 41 61 321 77 44
Fax: ++ 41 61 321 77 45

Dipl. Soz. Ina Otte
Institut für Hausarztmedizin und
Institut für Bioethik der Universität Basel
Bernoullistr. 28
CH 4056 Basel
Tel: ++ 41 61 267 17 82

Um die Formulare elektronisch auswerten zu können, müssen die Kästchen korrekt markiert werden :
Richtig: ● falsch: ⊕ falsch: ⊘ Bitte füllen Sie für **jede** Frage ein Antwortfeld aus.

0645417311

Zu Ihrer Person, Praxis und ärztlichen Tätigkeit

1. Sind Sie als Hausarzt tätig? ○ Ja ○ Nein

Wenn Sie nicht als Hausarzt/Hausärztin tätig sind, kreuzen Sie bitte nur die erste Frage an und schicken Sie uns den gesamten Fragebogen ohne die weiteren Fragen zu beantworten im vorfrankierten Kuvert zurück.

2. Geschlecht: ○ männlich ○ weiblich

3. Wie alt sind Sie?

○ unter 35 Jahre
○ 35 - 39 Jahre
○ 40 - 44 Jahre
○ 45 - 49 Jahre
○ 50 - 54 Jahre
○ 55 - 59 Jahre
○ 60 - 64 Jahre
○ über 65 Jahre

4. Wann haben Sie Ihr Staatsexamen gemacht?

○ vor 1960 ○ 1960 - 69 ○ 1970 - 79 ○ 1980 - 89 ○ 1990 - 99 ○ 2000 oder später

5. Wo haben Sie ihr Staatsexamen gemacht?

○ in der Deutschschweiz
○ in der Romandie
○ ausserhalb der Schweiz

6. Dauer der beruflichen Tätigkeit zwischen Staatsexamen und Praxiseröffnung:

○ unter 3 Jahre ○ 3 - 5 Jahre ○ 6 - 9 Jahre ○ 10 + Jahre

7. Wie lautet Ihr FMH-Titel (Haupttitel)?

○ Ich habe keinen FMH-Titel
○ Allgemeine Medizin
○ Innere Medizin
○ Allgemeine Innere Medizin
○ Anderes ______________________

○ Zusatztitel, Fähigkeitsnachweise: ______________________

8. Haben Sie eine spezifische Weiterbildung in Palliativmedizin absolviert?

○ Ja ○ Nein

Falls ja, welche: ______________________

Dauer der Weiterbildung: ______________________

9. Wo liegt Ihre Praxis?
Bitte geben Sie sowohl die Einwohnerzahl als auch die Region an.

Einwohnerzahl in Ihrer Ortschaft

- ○ bis 25.000 Einwohner
- ○ 25.000 bis 50.000 Einwohner
- ○ 50.000 bis 100.000 Einwohner
- ○ über 100.000 Einwohner

Region:
Bitte kreuzen Sie hier die für Ihren Praxisstandort am ehesten zutreffende Antwort an.
(**keine** Mehrfachnennung möglich)

- ○ Stadt
- ○ Agglomeration einer Stadt
- ○ Ländliche Region
- ○ Bergregion

10. In welcher Art von Praxis arbeiten Sie? (Mehrfachnennung möglich)

- ○ Einzelpraxis
- ○ Gruppenpraxis mit 2 - 3 Ärzten
- ○ Gruppenpraxis mit > 3 Ärzten
- ○ HMO-Praxis
- ○ Angeschlossen an ein Managed Care-System (Ärztenetzwerk)

11. Betreuen Sie neben Ihrer Praxistätigkeit auch Spitalpatienten/-patientinnen?

○ Ja ○ Nein

12. Wie üben Sie Ihre Tätigkeit aus?

- ○ Vorwiegend selbständig erwerbend
- ○ Vorwiegend angestellt

13. Zu wie vielen Stellenprozenten haben Sie *im Jahr 2013* durchschnittlich als Hausarzt/Hausärztin gearbeitet?

____ %

14. Wie viele Konsultationen halten Sie durchschnittlich pro Halbtag ab?

____ Konsultationen/Halbtag

15. Wie viele Besuche machen Sie durchschnittlich pro Monat (Hausbesuche, Alters-Pflegeheim, Behindertenheim etc.)?
(Falls Sie in einem Alters- und Pflegeheim im Rahmen eines Besuchs z.B. 10 Pensionäre sehen, geben Sie bitte **einen Besuch** und **zehn Konsultationen** an)

____ Besuche/Monat

____ Konsultationen/Monat

16. Wie viele Ihrer Patienten/Patientinnen sind im Jahr 2013 verstorben?

A. **Plötzlich oder nach sehr kurzer Krankheit,** also innert 1 bis 4 Wochen, ohne vorbestehende schwere Erkrankung, beispielsweise nach akuter Meningitis, Unfall.	○ 0	○ 1 - 5	○ 6 - 15	○ 16+
B. **Nach einer längeren Krankheit (über einen Monat),** beispielsweise nach Tumor, Demenz, COPD, Herzinsuffizienz, ALS.	○ 0	○ 1 - 5	○ 6 - 15	○ 16+

17. Bei wie viele Ihrer Patienten/Patientinnen, die im Jahr 2013 ...

**...nach einem Tumorleiden** verstorben sind, waren Sie **in den letzten 2 Wochen** ihres Lebens die medizinische Hauptbetreuungsperson?	○ 0	○ 1 - 5	○ 6 - 15	○ 16+
**...nach einem Nichttumorleiden** verstorben sind (wie beispielsweise COPD, Herzinsuffizienz, ALS, Demenz), waren Sie **in den letzten 2 Wochen** ihres Lebens die medizinische Hauptbetreuungsperson?	○ 0	○ 1 - 5	○ 6 - 15	○ 16+

18. Wie oft wurden Ihre Patienten/Patientinnen im Jahre 2013 *in den letzten 1 bis 3 Lebenswochen vor dem Tode* verlegt?

☐ gar nicht (bitte weiter zu Frage 25)

**A. Vom Pflegeheim**	sehr häufig	häufig	manchmal	selten	nie
ins Akut- oder Geriatriespital	○	○	○	○	○
nach Hause	○	○	○	○	○
in eine Palliativeinrichtung	○	○	○	○	○

B) **Vom Akut- oder Geriatriespital**	sehr häufig	häufig	manchmal	selten	nie
nach Hause	○	○	○	○	○
in ein Pflegeheim	○	○	○	○	○
in eine Palliativeinrichtung	○	○	○	○	○

C) **Von Zuhause**	sehr häufig	häufig	manchmal	selten	nie
in ein Akut- oder Geriatriespital	○	○	○	○	○
in ein Pflegeheim	○	○	○	○	○
in eine Palliativeinrichtung	○	○	○	○	○

19. Was waren Gründe für diese Verlegungen? (Bitte alle aufgeführten Gründe bewerten)

**Gründe**	sehr häufig	häufig	manchmal	selten	nie
Zu schwierige Symptomkontrolle	○	○	○	○	○
Zu kleines oder fehlendes Betreuungsnetz	○	○	○	○	○
Erschöpfung des Betreuungsnetzes	○	○	○	○	○
Wunsch des Patienten/der Patientin	○	○	○	○	○
Wunsch der Angehörigen	○	○	○	○	○
Fehlende Ressourcen der Hausärzte/-innen	○	○	○	○	○
Finanzielle Gründe	○	○	○	○	○

Qualität der Betreuung am Lebensende

20. Bitte geben Sie an, inwiefern die folgenden Aussagen für Sie zutreffen. Ich denke, dass ...

		trifft völlig zu	trifft über-wiegend zu	trifft teilweise zu	trifft eher nicht zu	trifft gar nicht zu
A.	… ich in meiner **Ausbildung** (Medizinstudium) zu Aspekten der Palliativmedizin ausreichend ausgebildet wurde.	○	○	○	○	○
B.	…. ich in meiner **Weiterbildung** (Zeit bis zum Facharzttitel) auf dem Gebiet der Palliativmedizin im Hinblick auf meine spätere hausärztliche Tätigkeit ausreichend vorbereitet wurde.	○	○	○	○	○
C.	… die **universitäre Ausbildung und eine internistische Weiterbildung** ausreichen, um später kompetent palliativmedizinisch tätig zu sein.	○	○	○	○	○
D.	… das Fortbildungsangebot in meiner Region auf dem Gebiet der Palliativmedizin ausreichend ist.	○	○	○	○	○

21. Welche Qualitätsmerkmale sind aus Ihrer Sicht für eine gute palliativmedizinische Versorgung wichtig? Bitte gewichten Sie die folgenden Qualitätsmerkmale aus Ihrer ganz persönlichen Perspektive.

		sehr wichtig	wichtig	teilweise wichtig	eher unwichtig	unwichtig
A.	Erkennen / Behandeln von Schmerzen	○	○	○	○	○
B.	Erkennen / Behandeln von Nausea, Erbrechen	○	○	○	○	○
C.	Erkennen / Behandeln von Obstipation, Ileus	○	○	○	○	○
D.	Erkennen / Behandeln von Unruhe, Delir	○	○	○	○	○
E.	Erkennen / Behandeln von Angst, Depression	○	○	○	○	○
F.	Ernährungsberatung	○	○	○	○	○
G.	Angehörigenbetreuung	○	○	○	○	○
H.	Umgang mit Verlust und Trauer	○	○	○	○	○
I.	Kommunikative Kompetenz	○	○	○	○	○
J.	Kompetenz in Entscheidungen am Lebensende	○	○	○	○	○
K.	Beratung bezüglich Patientenverfügungen	○	○	○	○	○
L.	Adäquater Umgang mit Suizidwünschen	○	○	○	○	○
M.	Adäquater Umgang mit Sterbewünschen	○	○	○	○	○
N.	Umgang mit spirituellen Bedürfnissen	○	○	○	○	○
O.	Palliative Betreuung von Menschen anderer Kulturkreise	○	○	○	○	○
P.	Adäquater Umgang mit der eigenen emotionalen Belastung	○	○	○	○	○
Q.	Vorwegnahme von Krisen (Antizipation)	○	○	○	○	○
R.	Bereitschaft zur Übernahme von Hausbesuchen und/oder Besuchen in Pflegeinstitutionen	○	○	○	○	○
S.	Telefonische Erreichbarkeit rund um die Uhr	○	○	○	○	○
T.	Koordination einer vernetzten Versorgung	○	○	○	○	○
U.	Weitere, nämlich:	○	○	○	○	○

4834417315

22. In welchen Bereichen fühlen Sie sich sicher beziehungsweise unsicher?						
	sehr sicher	sicher	teilweise sicher	eher unsicher	unsicher	kommt nicht vor
A. Erkennen / Behandeln von Schmerzen	○	○	○	○	○	○
B. Erkennen / Behandeln von Nausea, Erbrechen	○	○	○	○	○	○
C. Erkennen / Behandeln von Obstipation, Ileus	○	○	○	○	○	○
D. Erkennen / Behandeln von Unruhe, Delir	○	○	○	○	○	○
E. Erkennen / Behandeln von Angst, Depression	○	○	○	○	○	○
F. Ernährungsberatung	○	○	○	○	○	○
G. Pädiatrische Palliative Care	○	○	○	○	○	○
H. Geriatrische Palliativ Care	○	○	○	○	○	○
I. Angehörigenbetreuung	○	○	○	○	○	○
J. Umgang mit Verlust und Trauer	○	○	○	○	○	○
K. Kommunikation	○	○	○	○	○	○
L. Entscheidungen am Lebensende	○	○	○	○	○	○
M. Patientenverfügung	○	○	○	○	○	○
N. Umgang mit Suizidwünschen	○	○	○	○	○	○
O. Umgang mit Sterbewünschen	○	○	○	○	○	○
P. Umgang mit spirituellen Bedürfnissen	○	○	○	○	○	○
Q. Palliative Betreuung von Menschen anderer Kulturkreise	○	○	○	○	○	○
R. Umgang mit der eigenen emotionalen Belastung	○	○	○	○	○	○
S. Vorwegnahme von Krisen (Antizipation)	○	○	○	○	○	○
T. Koordination einer vernetzten Versorgung	○	○	○	○	○	○
U. Weitere:	○	○	○	○	○	○

23. Bitte bewerten Sie folgende Aussagen für Ihre Region:		trifft völlig zu	trifft überwiegend zu	trifft teilweise zu	trifft eher nicht zu	trifft gar nicht zu
A.	Die generelle Qualität der medizinischen Versorgung am Lebensende erachte ich als gut.	○	○	○	○	○
B.	Die palliativmedizinische Betreuung von sterbenden Patienten/Patientinnen zu Hause ist von guter Qualität.	○	○	○	○	○
C.	Die palliativmedizinische Betreuung von sterbenden Patienten/Patientinnen im Alters-/Pflegeheim ist von guter Qualität.	○	○	○	○	○
D.	Kranke Personen und Angehörige haben zu spät Zugang zur medizinischen Versorgung am Lebensende.	○	○	○	○	○
E.	Es bestehen genügend Versorgungsnetze, um Patienten/Patientinnen bis zum Lebensende zu Hause betreuen zu können.	○	○	○	○	○
F.	Die Mehrheit der Patienten/Patientinnen verstirbt in der von ihnen bevorzugten Umgebung (zu Hause, Spital, Alters-Pflegeheim, Palliativstation, Hospiz).	○	○	○	○	○
G.	Haus-, Spezial- und Spitalärzte ziehen bei Patienten /Patientinnen am Lebensende nicht immer am gleichen Strick.	○	○	○	○	○

Voraussetzungen für die Betreuung am Lebensende

24.Welche Gründe könnten Ihrer Meinung nach dazu führen, dass Sie einen Palliativpatienten/eine Palliativpatientin nicht mehr weiter betreuen und einen Kollegen / eine Kollegin (Palliativmediziner/Palliativmedizinerin, Spezialarzt/Spezialärztin) oder eine Institution anfragen, die Betreuung des Patienten zu übernehmen?

		trifft völlig zu	trifft überwiegend zu	trifft teilweise zu	trifft eher nicht zu	trifft gar nicht zu
A.	Zeitmangel	○	○	○	○	○
B.	Unbefriedigende Bezahlung	○	○	○	○	○
C.	Unvereinbarkeit mit anderen Verpflichtungen	○	○	○	○	○
D.	Weil andere Fachpersonen besser für diese Aufgabe ausgebildet sind.	○	○	○	○	○
E.	Weil ich keine Hausbesuche anbieten kann.	○	○	○	○	○
F.	Weil ich mich zu wenig unterstützt fühle.	○	○	○	○	○
G.	Weil die eigene emotionale Belastung zu gross ist	○	○	○	○	○

25. Inwiefern treffen folgende Aussagen auf Sie zu?	trifft völlig zu	trifft überwiegend zu	trifft teilweise zu	trifft eher nicht zu	trifft gar nicht zu
A. Ich empfinde die Betreuung und Begleitung von sterbenden Menschen und ihren Angehörigen als eine befriedigende Aufgabe.	○	○	○	○	○
B. Ich fühle mich durch die Betreuung von sterbenden Menschen emotional belastet.	○	○	○	○	○
C. Ich habe ausreichend Möglichkeiten, mich bei palliativmedizinischen Fragen mit Kollegen/ Kolleginnen zu besprechen (Balint-Gruppe, Qualitätszirkel).	○	○	○	○	○

Versorgungsstrukturen

26. Befindet sich im Einzugsgebiet Ihrer Praxis ein Hospiz, eine Palliativstation oder eine Palliativklinik?

○ Ja ○ Nein ○ Weiss ich nicht

27. Haben Sie die Möglichkeit, mit einer spezialisierten Spitex zusammenzuarbeiten (beispielsweise onkologische Spitex, mobiles Palliative Care Team)?

○ Ja ○ Nein ○ Weiss ich nicht

28. Nutzen Sie in Ihrem ärztlichen Alltag palliativmedizinische Konsilien (telefonisch oder am Krankenbett), beispielsweise mit spezialisierter Spitex, Palliativmediziner/-innen. Geriater/innen?

○ Ja

○ Bedarf vorhanden, aber Dienste nicht verfügbar

○ Meines Erachtens nicht notwendig

29. Inwiefern stimmen Sie folgende Aussagen zu?	stimme völlig zu	stimme überwiegend zu	stimme teilweise zu	stimme eher nicht zu	stimme gar nicht zu
A. Die medizinische Betreuung am Lebensende sollte generell den **Hausärzten/-ärztinnen** übertragen werden.	○	○	○	○	○
B. Die medizinische Betreuung am Lebensende sollte von den jeweils **involvierten Fachärzten/-ärztinnen** übernommen werden (zum Beispiel von Onkologen/Onkologinnen).	○	○	○	○	○
C. Die medizinische Betreuung am Lebensende sollte im Prinzip den **Hausärzten/-ärztinnen** übertragen werden. Wünschenswert ist die Möglichkeit der Unterstützung durch Palliativmediziner/-innen für schwierige Fälle.	○	○	○	○	○

30. Bitte bewerten Sie nachfolgende Aussagen aus Ihrer persönlichen Perspektive.	stimme zu	stimme eher zu	stimme teilweise zu	stimme eher nicht zu	stimme gar nicht zu
A. Angehörige übernehmen in der Versorgung am Lebensende wichtige psychosoziale und pflegerische Funktionen.	○	○	○	○	○
B. Eine umfassende palliativmedizinische Versorgung zu Hause bringt oft hohe Kosten für den Patienten/ die Patientin und seine/ihre Angehörigen mit sich.	○	○	○	○	○
C. Die finanzielle Belastung eines Patienten/ einer Patientin am Lebensende kann so hoch sein, dass eine Betreuung zu Hause nicht mehr möglich ist.	○	○	○	○	○
D. Angehörige und Patienten/Patientinnen sind bezüglich ihrer Möglichkeiten, finanzielle Unterstützungsangebote wahrzunehmen, gut informiert.	○	○	○	○	○
E. Angehörige und Patienten/Patientinnen sind bezüglich ihrer Möglichkeiten, ambulante Pflegeangebote wahrzunehmen, gut informiert.	○	○	○	○	○
F. Ein Casemanager/eine Casemanagerin sollte die Koordination der Dienstleistungen für Patienten/ Patientinnen am Lebensende übernehmen.	○	○	○	○	○

31. Meiner Erfahrung nach …	trifft völlig zu	trifft überwiegend zu	trifft teilweise zu	trifft eher nicht zu	trifft gar nicht zu
A. ...muss ich seit Einführung des Fallkostenpauschalen-Systems (DRG) in den Schweizer Spitälern vermehrt Patienten/Patientinnen am Lebensende zu Hause betreuen.	○	○	○	○	○
B. …kommt es seit Einführung des Fallkostenpauschalen-Systems (DRG) häufiger dazu, dass ich Patienten/Patientinnen am Lebensende kurz nach ihrer Entlassung wieder in eine Klinik überweisen muss.	○	○	○	○	○
C. …hat die Umstellung der Spitäler auf das Fallpauschalen-System keinen Einfluss auf den **Betreuungsort** meiner Patienten/Patientinnen am Lebensende.	○	○	○	○	○
D. …hat die Umstellung der Spitäler auf das Fallpauschalen-System keinen Einfluss auf die **Betreuungsqualität** meiner Patienten/Patientinnen am Lebensende.	○	○	○	○	○

2684417316

Zusammenarbeit mit anderen Medizinalpersonen und Nicht-Medizinalpersonen

32. Bitte gewichten Sie die Zusammenarbeit mit folgenden Akteuren bei der Versorgung von Palliativpatienten / - patientinnen. Geben Sie dabei an, welche Akteure Sie dabei als wichtig und welche Sie als eher weniger wichtig einstufen:

		sehr wichtig	wichtig	teilweise wichtig	eher unwichtig	unwichtig
A.	Für das Fachgebiet zuständige Spezialärzte/ärztinnen (z.B. aus der Onkologie, Neurologie)	○	○	○	○	○
B.	Allgemeine Spitex	○	○	○	○	○
C.	Spezialisierte Spitex z.B. onkologische Spitex	○	○	○	○	○
D.	Mobiles Palliative Care-Team (interdisziplinär, mindestens mit Palliativmediziner/in und spezialisierter Pflege)	○	○	○	○	○
E.	Palliativmediziner/in	○	○	○	○	○
F.	Pflegende Angehörige	○	○	○	○	○
G.	Akutspital in der Region	○	○	○	○	○
H.	Geriatriespital in der Region	○	○	○	○	○
I.	Alters-Pflegeheim	○	○	○	○	○
J.	Hospiz (Pflegeheimstatus)	○	○	○	○	○
K.	Palliativstation oder Palliativklinik	○	○	○	○	○
L.	Krebsliga	○	○	○	○	○
M.	Weitere Ligen und Patientenorganisationen für Demenz, Muskelerkrankungen, Rheuma u.a.	○	○	○	○	○
N.	Physiotherapeuten/innen	○	○	○	○	○
O.	Kunsttherapeuten (Musik-, Maltherapeuten)	○	○	○	○	○
P.	Seelsorger/ Seelsorgerinnen	○	○	○	○	○
Q.	Psychologen/Psychologinnen, Psychiater/innen	○	○	○	○	○
R.	Apotheker/innen	○	○	○	○	○
S.	Casemanager/innen	○	○	○	○	○

Entscheidungen am Lebensende

33. Bitte schätzen Sie, wie viele Ihrer Patienten/Patientinnen im Alter über 65 Jahren eine Patientenverfügung haben:

Anteil Patienten/Patientinnen über 65 Jahre mit Patientenverfügung

○ bis 25% ○ 26 - 50% ○ 51 - 75% ○ 75% +

34. In der Mehrzahl der Fälle wird das Thema Patientenverfügung angesprochen von …

- ○ mir
- ○ den Patienten/Patientinnen
- ○ den Angehörigen

35. Ich spreche mit meinen Patienten/Patientinnen in der Regel über eine Patientenverfügung, wenn…

		trifft völlig zu	trifft überwiegend zu	trifft teilweise zu	trifft eher nicht zu	trifft gar nicht zu
A.	sie noch gesund sind.	○	○	○	○	○
B.	sie schwer erkrankt sind.	○	○	○	○	○
C.	eine schwere Erkrankung fortschreitet.	○	○	○	○	○
D.	sich die letzte Lebensphase abzeichnet.	○	○	○	○	○

36. Ich erneuere Patientenverfügungen nach einer gewissen Zeit …

- ○ häufig (bei mehr als der Hälfte der Patienten/Patientinnen)
- ○ gelegentlich
- ○ selten
- ○ nie

37. Bitte bewerten Sie nachfolgende Aussagen aus Ihrer persönlichen Perspektive.

		trifft völlig zu	trifft überwiegend zu	trifft teilweise zu	trifft eher nicht zu	trifft gar nicht zu
A.	Patientenverfügungen sind für mich eine wesentliche Hilfe bei der Entscheidungsfindung am Lebensende.	○	○	○	○	○
B.	Ich wäre froh, wenn ich häufiger auf Patientenverfügungen zurückgreifen könnte.	○	○	○	○	○
C.	Patientenverfügungen sind hilfreich, weil sie den Angehörigen in Situationen am Lebensende Entscheidungsdruck abnehmen.	○	○	○	○	○

38. Bitte bewerten Sie nachfolgende Aussagen aus Ihrer persönlichen Perspektive.		trifft völlig zu	trifft überwiegend zu	trifft teilweise zu	trifft eher nicht zu	trifft gar nicht zu
A.	Vor Entscheidungen am Lebensende spreche ich mich mit den Angehörigen ab.	○	○	○	○	○
B.	Vor Entscheidungen am Lebensende spreche ich mich mit den involvierten Pflegenden der Spitex oder des Pflegeheims ab.	○	○	○	○	○
C.	Bei Entscheidungen am Lebensende im Spital werde ich mit einbezogen.	○	○	○	○	○
D.	Bei Entscheidungen am Lebensende im Spital würde ich als Hausarzt/Hausärztin gerne vermehrt mit einbezogen werden.	○	○	○	○	○

Umgang mit Suizidwünschen

39. Von wievielen Patienten / Patientinnen wurden Sie in den letzten 3 Jahren in einer konkreten Situation auf einen assistierten Suizid angesprochen?

_____ Anzahl Patienten/Patientinnen (Bitte Zahl angeben)

40. Bitte kreuzen Sie an, welche Aussage Ihrer eigenen Haltung am nächsten kommt:

A. Ich verstehe den Wunsch von Patienten/Patientinnen nach einem assistierten Suizid und ich würde sie dabei unterstützen und begleiten.	○
B. Ich verstehe den Wunsch von Patienten/Patientinnen nach einem assistierten Suizid, aber ich würde den Patienten/ die Patientin an eine Sterbehilfe-Organisation weiterweisen.	○
C. Ich bin generell gegen assistierten Suizid und erkläre meinen Patienten/Patientinnen, dass meine ärztliche Aufgabe darin besteht, Leiden zu verringern und Leben zu schützen.	○
D. Andere, nämlich ___________________	○

41. Bitte bewerten Sie nachfolgende Aussagen aus Ihrer persönlichen Perspektive.	trifft völlig zu	trifft überwiegend zu	trifft teilweise zu	trifft eher nicht zu	trifft gar nicht zu
A. Suizidwünsche meiner Patienten/ Patientinnnen sind für mich eine emotionale Belastung.	○	○	○	○	○
B. Suizidwünsche meiner Patienten/ Patientinnen verunsichern mich.	○	○	○	○	○
C. Assistierter Suizid kann berechtigt sein, um einen unkontrollierten Suizid zu verhindern.	○	○	○	○	○
D. Ich fühle mich kompetent, mit einem Suizidwunsch eines Patienten/ einer Patientin umzugehen.	○	○	○	○	○
E. Ich kann mir vorstellen, das Barbiturat-Präparat für assistierten Suizid zu verschreiben (NaP-Rezept).	○	○	○	○	○

Perspektive der Patienten/Patientinnen

42. Welche Aspekte sind aus Ihrer Sicht für Patienten/Patientinnen und deren Angehörige am Lebensende wichtig?	sehr wichtig	wichtig	teilweise wichtig	eher unwichtig	unwichtig
A. Symptomfreiheit	○	○	○	○	○
B. Korrekte Aufklärung	○	○	○	○	○
C. Mögliche Probleme und deren Lösungen frühzeitig zu besprechen	○	○	○	○	○
D. Von einem persönlichen Arzt/einer persönlichen Ärztin bis zum Tod begleitet zu werden.	○	○	○	○	○
E. Mit jemandem über Ängste sprechen zu können.	○	○	○	○	○
F. Keine zu grosse Belastung für Angehörige zu sein.	○	○	○	○	○
G. Entscheidungen am Lebensende selbst fällen zu können.	○	○	○	○	○
H. Beachten spiritueller Bedürfnisse.	○	○	○	○	○
I. Pflege- und Sterbeort selbst wählen können.	○	○	○	○	○
J. Beim Sterben nicht alleine zu sein.	○	○	○	○	○

2307417318

Tarifgestaltung und Honorierung

43. Bitte geben Sie an, wie weit Sie folgenden Aussagen zustimmen:

		stimme zu	stimme eher zu	stimme teilweise zu	stimme eher nicht zu	stimme gar nicht zu
A.	Palliativmedizinische ärztliche Leistungen für Patienten/Patientinnen zu Hause sowie im Alters- und Pflegeheim können adäquat verrechnet werden.	○	○	○	○	○
B.	Dass palliativmedizinische ärztliche Leistungen nicht adäquat verrechnet werden können, könnte zu einer geringeren Bereitschaft führen, die palliativ-medizinische Betreuung von Patienten/Patientinnen zu übernehmen.	○	○	○	○	○
C.	Rechnungen von verstorbenen Patienten/ Patientinnen werden oftmals nicht bezahlt.	○	○	○	○	○

Kommentare:

Vielen herzlichen Dank!

Senden Sie den Fragebogen nun bitte mit dem vorfrankierten Kuvert an folgende Adresse zurück:

Frau Dipl. Soz. Ina C. Otte
Institut für Hausarztmedizin IHAMB?
Universität Basel
Petersgraben 4
4031 Basel
