# Supplementary material for: General Practitioners’ Attitudes towards Essential Competencies in End-of-Life Care: A Cross-Sectional Survey
Source: PLoS One. 2017 Feb 1;12(2):e0170168. doi: 10.1371/journal.pone.0170168 (PMC5287469; doi:10.1371/journal.pone.0170168)
Supplement: S2 Appendix — (DOCX) [file pone.0170168.s002.docx]

**About you, your practice and your activities as a physician**

1. **Are you working as a GP?**

○ Yes ○ No

If you are not working as a GP, please mark only the first question without answering any other questions and send back the whole questionnaire using the post-paid envelope.

1. **Sex**

○ Male ○ Female

1. **How old are you?**

○ Under 35 years

○ 35 - 39 years

○ 40 - 44 years

○ 45 - 49 years

○ 50 – 54 years

○ 55 - 59 years

○ 60 - 64 years

○ Over 65

1. **Did you complete a vocational palliative care training?**

○ Yes ○ No

If yes, which one: ___________________________________________________________

Duration of vocational palliative care training: _____________________________________

1. **Where is your practice?**

**Please indicate the number of inhabitants as well as the region.**

**Number of inhabitant of your region**

○ Up to 25.000 inhabitants

○ 25.000 to 50.000 inhabitants

○ 50.000 to 100.000 inhabitants

○ Over 100.000 inhabitants

**Region:**

Please indicate the best answer concerning the location of your practice (no multiple answers possible).

○ City

○ Agglomeration of a city

○ Rural region

○ Mountain region

1. **In which type of practice do you work?**

(Multiple answers possible)

○ Single practice

○ Group practice with 2-3 GPs

○ Group practice with > 3 GPs

○ Health Maintenance Organisation practice

○ Associated with Managed Care-System (medical network)

1. **What was your average workload as a GP in 2013?**

_________________________________ %

1. **How many consultations do you usually have per half day?**

_______ consultations/half day

1. **How many home visits do you usually do per month? (Home visits, nursing home visits, home for the handicapped)**

____________ visits/month

____________ consultations/month

1. **How many patients of yours died in 2013?**

| 1. Suddenly or after a very short illness (within 1-4 days without pre-existing severe illness, e.g. after acute meningitis or diarrhoea) | ○ 0 ○ 1-5 ○ 6-15 ○ 16+ |
| --- | --- |
| 1. After a longer illness (more than a month, e.g. after tumour, dementia, COPD, heart insufficiency, ALS.) | ○ 0 ○ 1-5 ○ 6-15 ○ 16+ |

| 1. **Which quality criteria are important according to you for good palliative medicine? Please indicate following quality criteria from your personal view** | | | | | |
| --- | --- | --- | --- | --- | --- |
|  | Very important | Important | Partially important | Rather unimportant | unimportant |
| Recognition and treatment of pain |  |  |  |  |  |
| Recognition and treatment of nausea/vomitus |  |  |  |  |  |
| Recognition and treatment of constipation/ileus |  |  |  |  |  |
| Recognition and treatment of agitation/delirium |  |  |  |  |  |
| Recognition and treatment of anxiety/depression |  |  |  |  |  |
| Nutrition counselling |  |  |  |  |  |
| Care for the family |  |  |  |  |  |
| Cope with bereavement and grief |  |  |  |  |  |
| Communication |  |  |  |  |  |
| Competence in decisions at the end of life |  |  |  |  |  |
| Counselling regarding advanced directives |  |  |  |  |  |
| Adequate coping with suicidal wishes |  |  |  |  |  |
| Adequate coping with wishes to die |  |  |  |  |  |
| Cope with spiritual needs |  |  |  |  |  |
| Palliative care of people of different culture group |  |  |  |  |  |
| Cope with own emotional burden |  |  |  |  |  |
| Anticipation of crisis |  |  |  |  |  |
| Willingness to do home visits or visits in nurseries |  |  |  |  |  |
| Availability by telephone day and night |  |  |  |  |  |
| Coordination of networked health care |  |  |  |  |  |

| 1. **In which domains you feel confident or unconfident, respectively?** | | | | | | |
| --- | --- | --- | --- | --- | --- | --- |
|  | Very confident | Confident | Partially confident | Rather un-confident | Un-confident | Never occurs |
| Recognition and treatment of pain |  |  |  |  |  |  |
| Recognition and treatment of nausea/vomitus |  |  |  |  |  |  |
| Recognition and treatment of constipation/ileus |  |  |  |  |  |  |
| Recognition and treatment of agitation/delirium |  |  |  |  |  |  |
| Recognition and treatment of anxiety/depression |  |  |  |  |  |  |
| Nutrition counselling |  |  |  |  |  |  |
| Paediatric palliative care |  |  |  |  |  |  |
| Geriatric palliative care |  |  |  |  |  |  |
| Care for the family |  |  |  |  |  |  |
| Cope with bereavement and grief |  |  |  |  |  |  |
| Communication |  |  |  |  |  |  |
| Competence in decisions at the end of life |  |  |  |  |  |  |
| Counseling regarding advanced directives |  |  |  |  |  |  |
| Adequate coping with suicidal wishes |  |  |  |  |  |  |
| Adequate coping with wishes to die |  |  |  |  |  |  |
| Cope with spiritual needs |  |  |  |  |  |  |
| Palliative care of people of different culture group |  |  |  |  |  |  |
| Cope with own emotional burden |  |  |  |  |  |  |
| Anticipation of crisis |  |  |  |  |  |  |
| Coordination of networked health care |  |  |  |  |  |  |
